# MOSFET-Based Voltage Reference Circuits in the Last Decade: A Review

**DOI:** 10.3390/mi15121504

**Published:** 2024-12-17

**Authors:** Elisabetta Moisello, Edoardo Bonizzoni, Piero Malcovati

**Affiliations:** Department of Electrical, Computer and Biomedical Engineering, University of Pavia, 27100 Pavia, Italy; edoardo.bonizzoni@unipv.it (E.B.); piero.malcovati@unipv.it (P.M.)

**Keywords:** CMOS, MOSFET, voltage reference, temperature compensation, low power, low voltage, temperature coefficient, line sensitivity, PSR, bandgap, BJT, microelectronics

## Abstract

Voltage reference circuits are a basic building block in most integrated microsystems, covering a wide spectrum of applications. Hence, they constitute a subject of great interest for the entire microelectronics community. MOSFET-based solutions, in particular, have emerged as the implementation of choice for realizing voltage reference circuits, given the supply voltage scaling and the ever-lower power consumption specifications in various applications. For these reasons, this paper aims to review MOSFET-based voltage reference circuits, illustrating their principles of operation, as well as presenting a detailed overview of the state-of-the-art, in order to paint an accurate picture of the encountered challenges and proposed solutions found in the field in the last decade, thus providing a starting point for future research in the field.

## 1. Introduction

Voltage references have always constituted an essential building block in integrated circuits and microsystems, including data converters, DC–DC converters, linear regulators, oscillators, transducer interfaces, bio-implants, communication systems and RF modules, and wearable and portable devices. Voltage references should provide a stable DC voltage, ideally not affected by variations in process, supply voltage and temperature (PVT). In order to quantify the robustness of a voltage reference circuit against PVT variations, the main parameters typically considered are output voltage (i.e., reference voltage) spread, power supply rejection (PSR), line sensitivity (LS), and temperature coefficient (TC). The output voltage spread is usually calculated as

(1) $$\frac{\sigma }{\mu }\cdot 100\;\;\;\left[\%\right]$$

where σ and μ are the reference voltage standard deviation and mean value, derived when considering a sufficiently large sample population, in order to take into account the impact of process and mismatch variations.

In order to evaluate the circuit robustness against voltage supply variations, the PSR and the LS parameters are considered. The PSR quantifies the effect of supply voltage ripple variations on the implemented reference voltage and is calculated as

(2) $$PSR=20\cdot log_{10}\left|\frac{V_{REF}\left(f\right)}{V_{IN}\left(f\right)}\right|\;\;\;\left[dB\right]$$

where $V_{REF}\left(f\right)$ and $V_{IN}\left(f\right)$ are the reference voltage and supply voltage, respectively, derived considering an *f*-frequency sinusoidal small signal superimposed to the DC supply voltage.

The LS, also referred to as line regulation, is the ability of the voltage reference circuit to maintain a constant output voltage despite changes to the input current; it is defined as

(3) $$LS=\frac{\Delta V_{REF}}{\Delta V_{IN}\cdot V_{REF}}\cdot 100\;\;\;\left[\%/V\right]$$

where $\Delta V_{REF}$ is the reference voltage variation, obtained when considering supply voltage variation $\Delta V_{IN}$, which typically corresponds to the difference between the maximum and minimum supported supply voltages.

The TC provides a measure of the obtained reference voltage stability across the temperature range of interest and is defined as

(4) $$TC=\frac{V_{REF,\;max}-V_{REF,\;min}}{V_{REF,\;nominal}\left(T_{max}-T_{min}\right)}\cdot 10^{6}\;\;\;\left[\mathrm{ppm}/{}^{\circ}C\right]$$

where $V_{REF,\;nominal}$ is the nominal reference voltage, $V_{REF,\;max}$ and $V_{REF,\;min}$ are the maximum and minimum reference voltage values obtained across the considered temperature range, and $T_{max}$ and $T_{min}$ are the maximum and minimum considered temperatures, respectively, expressed in Celsius degrees.

In the last decade, thanks to the growing inclusion of electronic devices into our daily routine according to the desire to enable an increasingly “smarter” lifestyle, Internet of Things (IoT) devices, wireless sensor nodes, portable, wearable, and implantable electronics, as well as battery or energy-harvesting operated applications, have faced a growing demand [1]. All these types of devices typically require at least one voltage reference circuit, that, in addition to immunity to PVT variations, must satisfy the low-power and low-voltage requirements imposed by the need to extend the battery life and provide the desired operation even under the low voltage supply enabled by energy harvesting [2,3,4].

**Figure 1:**
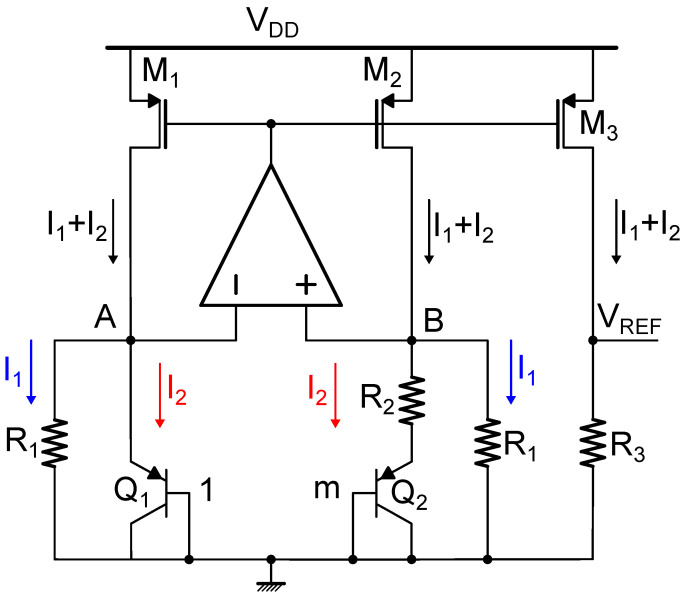
Schematic representation of a standard current-mode bandgap reference circuit.

Bandgap voltage references, based on BJT devices, have always constituted the most widespread solution for implementing high-performance (i.e., featuring low spread, PSR, LS, and TC parameters) voltage references. Bandgap circuits implement reference voltage $V_{REF}$ as the weighted sum of a proportional-to-absolute-temperature (PTAT) and a complementary-to-absolute-temperature (CTAT) component which compensate each other at the first order, thus providing a temperature-independent voltage. The basic bandgap voltage reference circuit [5] is illustrated in Figure 1. Its operation is based on BJT devices $Q_{1}$ and $Q_{2}$, which are of the same type but feature different emitter areas according to the factor *m*, on current mirrors $M_{1}-M_{2}-M_{3}$, on same-type resistors $R_{1}-R_{2}-R_{3}$, and on the operational amplifier action. In the illustrated schematic, pnp devices are employed since they are the most commonly used as they can be found in the form of parasitic vertical devices in any standard CMOS technology [6]; however, an analogous circuit architecture can be implemented also relying on npn BJTs, which can be found when the process supports p-wells. In the reported example, $M_{1}$, $M_{2}$, and $M_{3}$ are supposed to be matched and feature equal size; however, the mirroring factor could be exploited as an additional degree of freedom in the circuit design. Thanks to the operational amplifier action, which must provide sufficient open loop gain, nodes *A* and *B* are virtually short circuited and equal to $V_{EB,1}$, that is the emitter-to-base voltage of $Q_{1}$; hence, current $I_{1}$ is equal to $\frac{V_{EB,1}}{R_{1}}$, whereas current $I_{2}$ flowing through $R_{2}$ is given by $\frac{V_{EB,1}-V_{EB,2}}{R_{2}}$, where $V_{EB,2}$ is the emitter-to-base voltage of $Q_{2}$. Relying on the pnp BJT collector current expression

(5) $$I_{C}=A\;I_{SS}\;e^{\frac{V_{EB}}{V_{T}}}=A\;I_{SS}\;e^{\frac{qV_{EB}}{kT}}$$

where *A* is the emitter area, $I_{SS}$ the BJT saturation current, $V_{T}$ the thermal voltage, *q* the module of the electron charge, *k* the Boltzmann constant, and *T* the temperature expressed in Kelvin degrees, $V_{EB}$ can be derived as

(6) $$V_{EB}=V_{T}\;ln\left(\frac{I_{C}}{A\;I_{SS}}\right)$$

Hence,

(7) $$V_{EB,1}-V_{EB,2}=V_{T}\;ln\left(\frac{I_{2}}{A_{1}\;I_{SS}}\right)-V_{T}\;ln\left(\frac{I_{2}}{A_{2}\;I_{SS}}\right)=V_{T}\;ln\left(\frac{I_{2}\;A_{2}\;I_{SS}}{A_{1}\;I_{SS}\;I_{2}}\right)=V_{T}\;ln\left(m\right)$$

since the area of $Q_{2}$ ($A_{2}$) is *m* times the one of $Q_{1}$ ($A_{1}$). Currents $I_{1}$ and $I_{2}$ are summed at nodes *A* and *B*, giving rise to current $I_{1}+I_{2}$ that is mirrored to the output branch, thus generating the reference voltage $V_{REF}$ as

(8) $$V_{REF}=R_{3}\left(I_{1}+I_{2}\right)=R_{3}\left[\frac{V_{EB,1}}{R_{1}}+\frac{V_{T}ln\left(m\right)}{R_{2}}\right]=\frac{R_{3}}{R_{1}}\;V_{EB,1}+\frac{R_{3}}{R_{1}}\;V_{T}\;ln\left(m\right)$$

Since $V_{EB,1}$ and $V_{T}$ feature, respectively, a CTAT and a PTAT behaviour, by appropriately sizing the resistances it is possible to achieve temperature compensation at the first order and, consequently, very low TC. In order to further decrease, and therefore, improve, the TC value, additional temperature compensation circuits can be added for implementing curvature compensation [7], thus canceling higher order temperature dependencies.

Although bandgap reference circuits exhibit good performance and still constitute a topic of interest in research [8,9,10,11,12], they suffer from significant limitations in terms of minimum operating supply voltage and power consumption. The $V_{EB}$ (for pnp) or $V_{BE}$ (for npn) voltage is approximately 0.6–0.7 V, thus clearly limiting the minimum usable supply and implementable reference voltage; furthermore, the minimum achievable power consumption is of the order of a few microwatts, since, due to area and matching constraints, the integrated resistor cannot exceed values of few megaohms. Therefore, it is evident that traditional BJT-based voltage reference circuits cannot address the requirements of the battery and energy harvesting operated applications that have become more and more widespread in the last decade. Thankfully, BJT bandgap references are not the only implementation for voltage reference circuits, MOSFET-based structures have emerged as solutions for addressing low-voltage and very low-power specifications. With respect to BJT-based solutions; however, MOSFET-based implementations are typically more sensitive to process and temperature variations [6], hence, additional challenges are faced in their design. Given the current focus on applications featuring this type of requirements, in the frame of the growing inclusion of electronics in our daily life (e.g., IoT, portable, wearable, and even implantable devices), this review will concentrate on MOSFET-based voltage reference circuits, describing the underlying theoretical principles and summarizing the various solutions proposed in the last ten years.

This review paper is organized as follows: Section 2 outlines the theoretical principles behind MOSFET-based voltage references, Section 3 provides an overview of the various implementations proposed in the last decade, and Section 4 concludes the paper.

## 2. MOSFET-Based Voltage References: Principles of Operation

Several different ways for implementing MOSFET-based voltage references have been proposed in the literature; however, they can be mostly classified into two main topologies: bandgap-like and threshold voltage-based voltage reference circuits. In addition to these main types, other implementations can also be found (e.g., combining bandgap-like and threshold voltage-based topologies). These will be discussed more in detail in Section 3.

### 2.1. Bandgap-like Voltage References

The most straightforward way of implementing a MOSFET-based voltage reference stems directly from the discussed bandgap reference. The so-called bandgap-like voltage reference circuit employs the architectures developed for BJT-based solutions while substituting BJTs with MOSFET devices operating in the subthreshold region, whose governing equations closely resemble the ones describing the BJT operation. For instance, an NMOS transistor operating in the subthreshold region, has a drain-to-source current equal to

(9) $$I_{DS}=I_{D0}\;e^{\frac{q(V_{GS}-V_{TH})}{nkT}}\left(1-e^{\frac{-qV_{DS}}{kT}}\right)$$

where $I_{D0}$ is the drain current for $V_{GS}=V_{TH}$, $V_{GS}$ the gate-to-source voltage, $V_{TH}$ the threshold voltage, $V_{DS}$ the drain-to-source voltage, *q* the magnitude of the electron charge, *k* the Boltzmann constant, *T* the temperature expressed in Kelvin degrees, and *n* (subthreshold slope factor) a process-dependent parameter. $I_{D0}$ is equal to $\mu \frac{W}{L}C_{ox}\left(n-1\right)V_{T}^{2}$, where μ is the mobility, *W* the transistor width, *L* its channel length, $C_{ox}$ the gate capacitance per unit area, and $V_{T}$ the thermal voltage. For $V_{DS}>4V_{T}$ the expression can be simplified to

(10) $$I_{DS}=I_{D0}\;e^{\frac{q(V_{GS}-V_{TH})}{nkT}}$$

which closely matches the BJT collector current expression (5). From (10) the $V_{GS}$ can be derived as

(11) $$V_{GS}=V_{TH}+\frac{nkT}{q}ln\left(\frac{I_{DS}}{I_{D0}}\right)$$

that yields an overall CTAT behavior at the first order, similar to the BJT $V_{BE}$ or $V_{EB}$.

**Figure 2:**
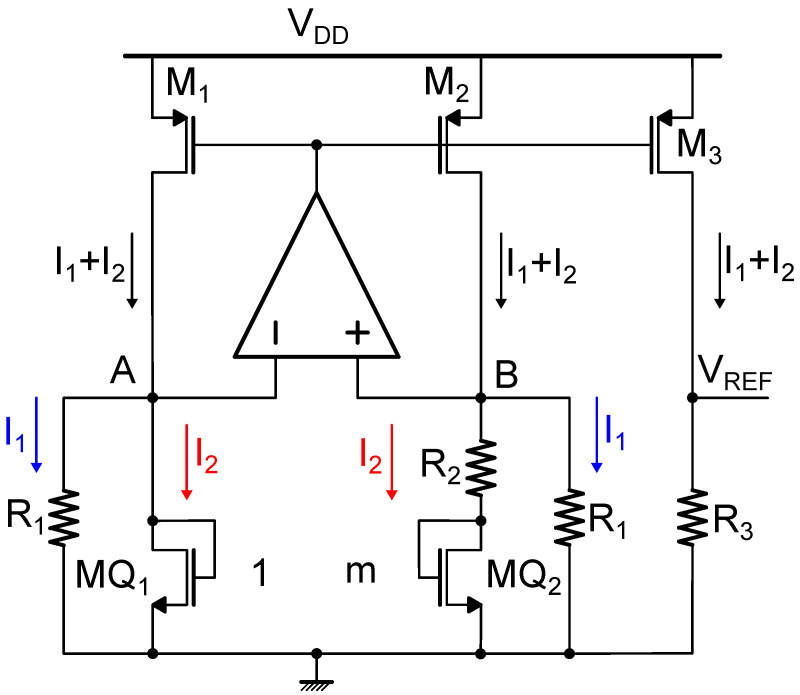
Schematic representation of a bandgap-like MOSFET-based reference circuit.

The standard bandgap-like voltage reference circuit, analogous to the BJT counterpart reported in Figure 1. As illustrated in Figure 2, the BJT devices $Q_{1}$ and $Q_{2}$ are substituted by diode-connected NMOS devices $MQ_{1}$ and $MQ_{2}$, where $MQ_{2}$ is composed by *m* transistors equal to $MQ_{1}$ placed in parallel. The reference voltage $V_{REF}$ is given by

(12) $$V_{REF}=R_{3}\left(I_{1}+I_{2}\right)=R_{3}\left(\frac{V_{GS,1}}{R_{1}}+\frac{V_{GS,1}-V_{GS,2}}{R_{2}}\right)$$

where (for i=1,2)

(13) $$V_{GS,i}=V_{TH,i}+\frac{nkT}{q}ln\left(\frac{I_{2}}{I_{D0,i}}\right)$$

and, given the different transistor sizes,

(14) $$I_{D0,2}=mI_{D0,1}$$

Hence,

(15) $$V_{REF}=R_{3}\left(\frac{V_{GS,1}}{R_{1}}+\frac{V_{GS,1}-V_{GS,2}}{R_{2}}\right)=\frac{R_{3}}{R_{1}}V_{GS,1}+\frac{R_{3}}{R_{2}}\frac{nkT}{q}ln\left(m\right)$$

supposing, as it is reasonable, that $V_{TH,1}=V_{TH,2}$. In this way, by appropriately sizing the resistances, the obtained $V_{REF}$ can be made independent from the temperature at the first order as $V_{GS,1}$ exhibits a CTAT behavior while $\frac{nkT}{q}ln\left(m\right)$ is PTAT.

The circuit shown in Figure 2 is not the sole realization of a bandgap-like MOSFET-based voltage reference, the different implementations can be found in the literature, and it will be discussed in more detail in Section 3. In general, all bandgap-like topologies rely on the action of an operational amplifier and two MOS devices operating in the subthreshold region for generating two currents with opposite temperature coefficients, which are then appropriately weighted and summed, and finally converted to a voltage, thus obtaining the desired $V_{REF}$, which is independent from temperature at the first order. As the temperature dependence, due to non-linearity, features also higher order terms, and additional compensation techniques have to be implemented to further lower the TC [6].

### 2.2. Threshold Voltage-Based Voltage References

Threshold voltage-based topologies, contrary to bandgap-like references, rely on the specific characteristics of MOSFET devices for implementing the desired reference voltage. In particular, as the name suggests, the transistor threshold voltage (or the difference between two MOS threshold voltages) is exploited. Differently from bandgap-like implementations, no operational amplifier is required, hence, this solution is particularly well suited for very low-power and low-voltage applications.

The most general threshold voltage-based reference circuit model consists of a current generator and a load. If the current generator is directly implemented through the voltage reference circuit, without the need for additional biasing, the reference circuit is referred to as self-biased.

**Figure 3:**
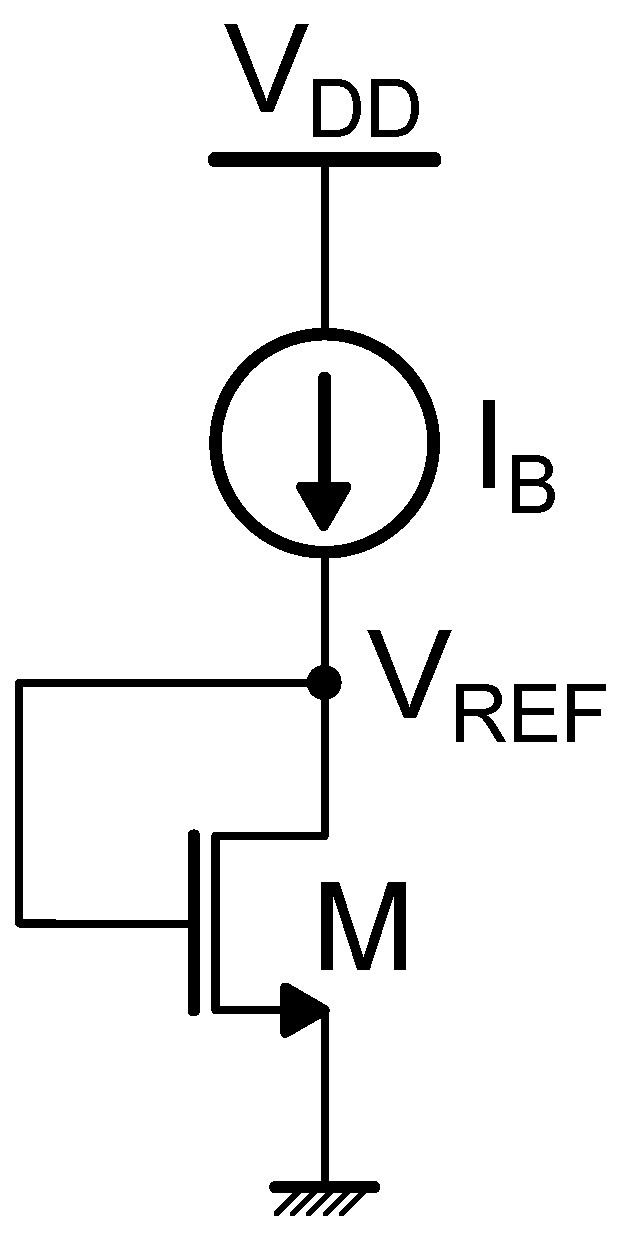
Schematic representation of a generic threshold voltage-based MOSFET reference circuit.

Different approaches exist to implement a temperature-independent $V_{REF}$ according to the general current generator and active load schematic. One possibility relies on exploiting the zero temperature coefficient (ZTC) operating point of a MOS transistor. In this case, the active load is typically replaced by a diode-connected NMOS device, as shown in Figure 3. The dual architecture relying on a diode-connected PMOS transistor is also possible [13]. The ZTC condition [14,15,16] corresponds to a specific biasing point where mutual compensation of the temperature dependencies of threshold voltage and carrier mobility occurs. Indeed, the threshold voltage $V_{TH}$ and the mobility μ can be approximately modeled as

(16) $$V_{TH}\left(T\right)=V_{TH}\left(T_{0}\right)+\alpha_{V_{TH}}\left(T-T_{0}\right)$$

(17) $$\mu \left(T\right)=\mu \left(T_{0}\right){\left(\frac{T}{T_{0}}\right)}^{\alpha_{\mu }}$$

where $T_{0}$ is a reference temperature (usually 300 K) and $\alpha_{V_{TH}}$ and $\alpha_{\mu }$ are technology dependent temperature coefficients, which commonly lie in the range of −3 mV/K to −0.5 mV/K and −2.42 to −1.2, respectively. Now, supposing transistor *M* is operating in saturation in strong inversion, its drain-to-source current can be approximated with

(18) $$I_{DS}\left(T\right)=\frac{1}{2}\frac{W}{L}C_{ox}\mu \left(T\right){\left(V_{GS}-V_{TH}\left(T\right)\right)}^{2}$$

where *W* is the transistor width, *L* its length, and $C_{ox}$ the gate capacitance per unit area. Substituting (16) and (17) into (18), differentiating with respect to the temperature and solving for the gate-to-source voltage where this temperature dependence becomes zero, one finds

(19) $$V_{GS0}=V_{TH}\left(T_{0}\right)+\alpha_{V_{TH}}T\left(1+\frac{2}{\alpha_{\mu }}\right)-\alpha_{V_{TH}}T_{0}.$$

**Figure 4:**
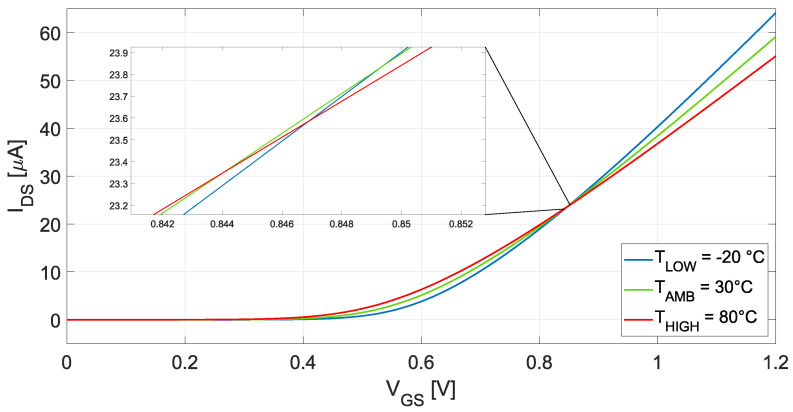
Graphical representation of a transistor ZTC point.

Provided that $\alpha_{\mu }=-2$, a temperature independent voltage

(20) $$V_{GS0}=V_{TH}\left(T_{0}\right)-\alpha_{V_{TH}}T_{0}$$

corresponding to $V_{REF}$ exists. While $\alpha_{\mu }=-2$ is a reasonable approximation, its value actually varies within a certain range, as explained before, hence, in more realistic scenarios, a single ZTC point does not exist and the simple (20) expression provides an approximate solution. Indeed, when plotting the drain-to-source current as a function of the gate-to-source voltage of a transistor for different temperatures, as shown in Figure 4, a single intercept point is not found. However, a group of points with the same $I_{DS}$ but slightly different $V_{GS}$ can be found, hence instead of a single $V_{GS0}$, there exists a range of gate-to-source voltages for which the drain current has a very low temperature coefficient. Although a zero-TC cannot be achieved, TCs in the order of 20 ppm/°C can still be obtained with this technique [15]. It is evident from (20) that the ZTC voltage is mainly determined by the threshold voltage, which can yield a reference voltage too high when low-voltage and low-power operation is targeted. To solve this issue, the transistor can be biased slightly outside of its ZTC point and an adequate bias current can be employed for canceling out the temperature dependency. The temperature behavior of the gate-to-source voltage of a MOS transistor biased slightly outside its ZTC point can be approximated by a simple linear model [17]:

(21) $$V_{GS}\left(T\right)\approx V_{TH}\left(T_{0}\right)+\alpha_{V_{GS}}T$$

where the sign of $\alpha_{V_{GS}}$ can be made positive or negative by biasing the transistor with a current, respectively, slightly above or slightly below its ZTC current value, or, given a fixed current, by varying the transistor aspect ratio. Hence, if the transistor is biased in its CTAT range with a negative $\alpha_{V_{GS}}$, it will feature a $V_{GS}$ voltage lower than its ZTC voltage; if then a PTAT biasing current is used, the two temperature dependencies will compensate each other, resulting in a reference voltage approximately constant with temperature [17]. The derived equations are valid for a saturated MOS device operating in strong inversion; however, the ZTC operating point can often be found in the moderate inversion region as well [17]; therefore, the $V_{GS0}$ can be derived relying on the EKV (Enz, Krummenacher, Vittoz) [17,18] or the Advanced Compact MOSFET (ACM) [19,20] model, while always supposing $\alpha_{\mu }=-2$. Analogously to the strong inversion case, a range of $V_{GS}$ ensuring low TC, rather than a single $V_{GS0}$ enabling zero-TC, is found.

**Figure 5:**
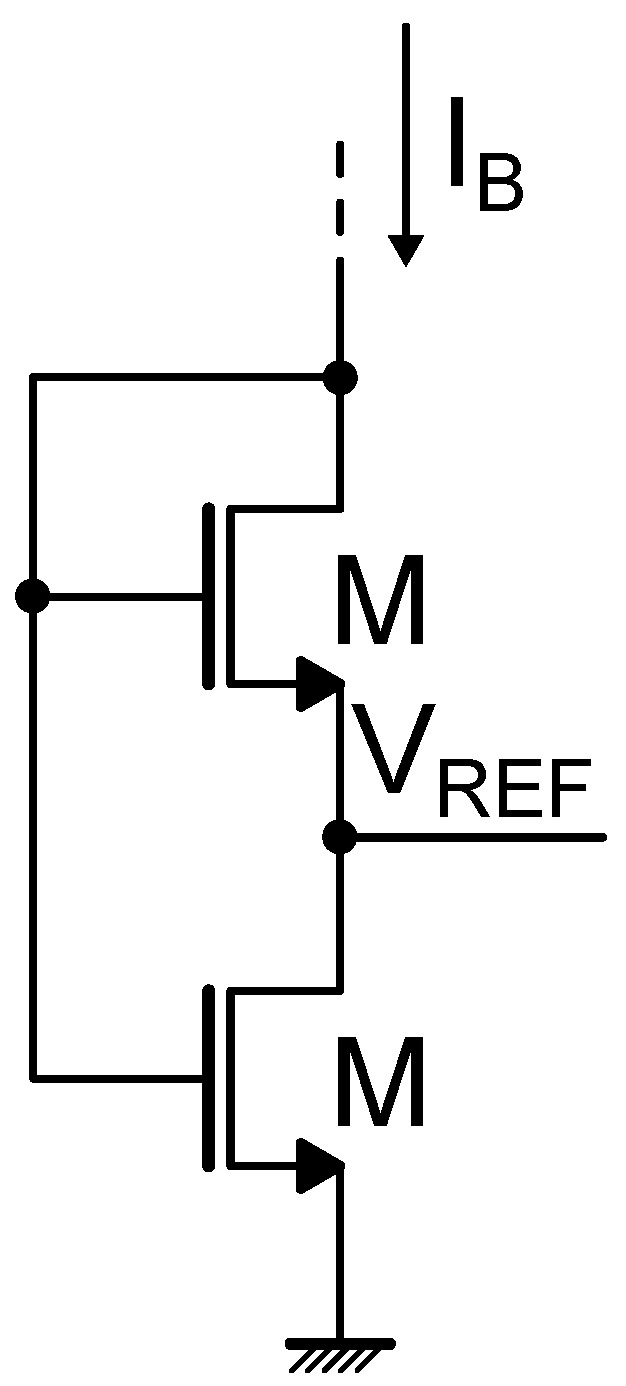
Schematic representation of a self-cascode MOSFET (SCM).

In addition to relying on the ZTC condition of a transistor, another way of implementing a threshold voltage-based reference exploits the difference between the threshold voltages of two MOS devices operating in subthreshold region; these types of circuits are referred to as $\Delta V_{TH}$-based voltage references. In this scenario, the most common topology employs the so-called self-cascode MOSFET (SCM) [21,22], which is illustrated in Figure 5. For proper operation, $M_{1}$ and $M_{2}$ are in weak inversion, with $M_{1}$ that can be either in triode or in saturation, whereas $M_{2}$ is always saturated [22]. The reported example considers NMOS devices for implementing the SCM structure, as it is the most common; however, also PMOS-based SCM architectures can be realized [23]. Supposing that both $M_{1}$ and $M_{2}$ are saturated (their $V_{DS}$ is larger than $4V_{T}$), their gate-to-source voltage can be expressed according to (13) as

(22) $$V_{GS,1}=V_{TH,1}+\frac{nkT}{q}ln\left(\frac{I_{B}}{I_{D0,1}}\right)$$

(23) $$V_{GS,2}=V_{TH,2}+\frac{nkT}{q}ln\left(\frac{I_{B}}{I_{D0,2}}\right)$$

The resulting reference voltage $V_{REF}$ is equal to

(24) $$V_{REF}=V_{GS,1}-V_{GS,2}=V_{TH,1}-V_{TH,2}+\frac{nkT}{q}ln\left(\frac{I_{D0,2}}{I_{D0,1}}\right)=\Delta V_{TH}+\frac{nkT}{q}ln\left(\frac{K_{2}I_{0,2}}{K_{1}I_{0,1}}\right)$$

where $K_{1}$ and $K_{2}$ are the aspect ratios of $M_{1}$ and $M_{2}$, respectively, and $I_{0,i}=\mu_{i}C_{ox,i}\left(n_{i}-1\right)V_{T}^{2}$. Given the threshold voltage model reported in (16), $\Delta V_{TH}$ can be expressed as

(25) $$\Delta V_{TH}=V_{TH,1}\left(T_{0}\right)-V_{TH,2}\left(T_{0}\right)+\left(\alpha_{V_{TH,1}}-\alpha_{V_{TH,2}}\right)\left(T-T_{0}\right).$$

Hence, substituting (25) into (24), the following expression is obtained:

(26) $$V_{REF}=V_{TH,1}\left(T_{0}\right)-V_{TH,2}\left(T_{0}\right)+\left(\alpha_{V_{TH,1}}-\alpha_{V_{TH,2}}\right)\left(T-T_{0}\right)+\frac{nkT}{q}ln\left(\frac{K_{2}I_{0,2}}{K_{1}I_{0,1}}\right)$$

By selecting the appropriate transistor type (e.g., thin-oxide, thick-oxide or native) among the ones offered by the selected technological process and by properly sizing $M_{1}$ and $M_{2}$, the temperature dependencies of the different terms making up $V_{REF}$ can be compensated. In particular, $V_{REF}$ is determined by three terms: since $V_{TH,1}\left(T_{0}\right)>V_{TH,2}\left(T_{0}\right)$, $V_{TH,1}\left(T_{0}\right)-V_{TH,2}\left(T_{0}\right)$ constitutes a positive voltage independent from temperature, whereas $\left(\alpha_{V_{TH,1}}-\alpha_{V_{TH,2}}\right)$ is usually negative, determining a CTAT term, and $\frac{nkT}{q}ln\left(\frac{K_{2}I_{0,2}}{K_{1}I_{0,1}}\right)$ is typically PTAT. By compensating the CTAT and PTAT terms a stable reference voltage with respect to temperature, at the first order, is obtained.

**Figure 6:**
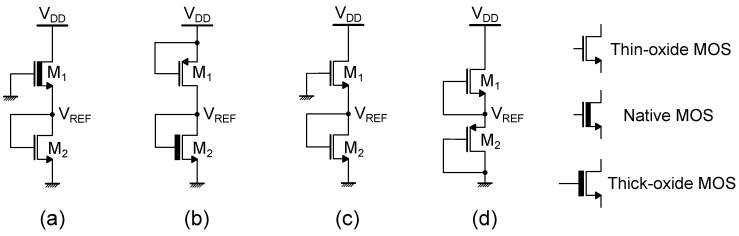
Schematic representation of a few 2T voltage reference topologies: (**a**) employs thin-oxide and native devices, (**b**) uses thick-oxide and thin-oxide devices, (**c**) relies on same-type devices, while (**d**) uses NMOS and PMOS devices.

In addition to SCM-based topologies, the so-called $\Delta V_{TH}$-based references can be implemented with simple 2-transistors (2T) structures where the devices are operated in subthreshold region. These implementations are particularly apt for ultra low-power consumption as they exploit the transistor leakage current for biasing. Different 2T topologies can be found [24,25,26,27], and a few examples are illustrated in Figure 6. Considering, for instance, the circuit shown in Figure 6a and supposing that both transistors are operated in weak inversion and are saturated ($V_{DS}>4V_{T}$), the drain-to-source currents of $M_{1}$ and $M_{2}$ are

(27) $$I_{DS,1}=\mu_{1}C_{ox,1}\frac{W_{1}}{L_{1}}\left(n_{1}-1\right)V_{T}^{2}\;e^{\frac{0-V_{REF}-V_{TH1}}{n_{1}V_{T}}}$$

(28) $$I_{DS,2}=\mu_{2}C_{ox,2}\frac{W_{2}}{L_{2}}\left(n_{2}-1\right)V_{T}^{2}\;e^{\frac{V_{REF}-0-V_{TH2}}{n_{2}V_{T}}}.$$

Currents $I_{DS,1}$ and $I_{DS,2}$ are equal, hence

(29) $$\mu_{1}C_{ox,1}\frac{W_{1}}{L_{1}}\left(n_{1}-1\right)V_{T}^{2}\;e^{\frac{0-V_{REF}-V_{TH1}}{n_{1}V_{T}}}=\mu_{2}C_{ox,2}\frac{W_{2}}{L_{2}}\left(n_{2}-1\right)V_{T}^{2}\;e^{\frac{V_{REF}-0-V_{TH2}}{n_{2}V_{T}}}$$

(30) $$\frac{\mu_{1}C_{ox,1}\frac{W_{1}}{L_{1}}\left(n_{1}-1\right)V_{T}^{2}}{\mu_{2}C_{ox,2}\frac{W_{2}}{L_{2}}\left(n_{2}-1\right)V_{T}^{2}}=e^{\frac{V_{REF}-V_{TH2}}{n_{2}V_{T}}+\frac{V_{REF}+V_{TH1}}{n_{1}V_{T}}}$$

(31) $$ln\left(\frac{\mu_{1}C_{ox,1}\frac{W_{1}}{L_{1}}\left(n_{1}-1\right)}{\mu_{2}C_{ox,2}\frac{W_{2}}{L_{2}}\left(n_{2}-1\right)}\right)=\frac{n_{1}\left(V_{REF}-V_{TH2}\right)+n_{2}\left(V_{REF}+V_{TH1}\right)}{n_{1}n_{2}V_{T}}.$$

Therefore, the reference voltage $V_{REF}$ can be derived as

(32) $$V_{REF}=\frac{n_{1}n_{2}V_{T}}{n_{1}+n_{2}}ln\left(\frac{\mu_{1}C_{ox,1}\frac{W_{1}}{L_{1}}\left(n_{1}-1\right)}{\mu_{2}C_{ox,2}\frac{W_{2}}{L_{2}}\left(n_{2}-1\right)}\right)+\frac{n_{2}V_{TH1}-n_{1}V_{TH2}}{n_{1}+n_{2}}$$

Considering the threshold voltage and mobility models described by (16) and (17), respectively, and imposing $\frac{\partial V_{\mathrm{REF}}}{\partial T}=0$, a condition on the sizing of the two transistors for ensuring temperature compensation can be found. The same method, using the circuit shown in Figure 6a, can be applied to any kind of 2T voltage reference topology and can be expanded also to the case of transistors that are not saturated ($V_{DS}<4V_{T}$), provided that the $I_{DS}$ expression is modified accordingly to take into account the effect of $V_{DS}$ (see (9)).

**Figure 7:**
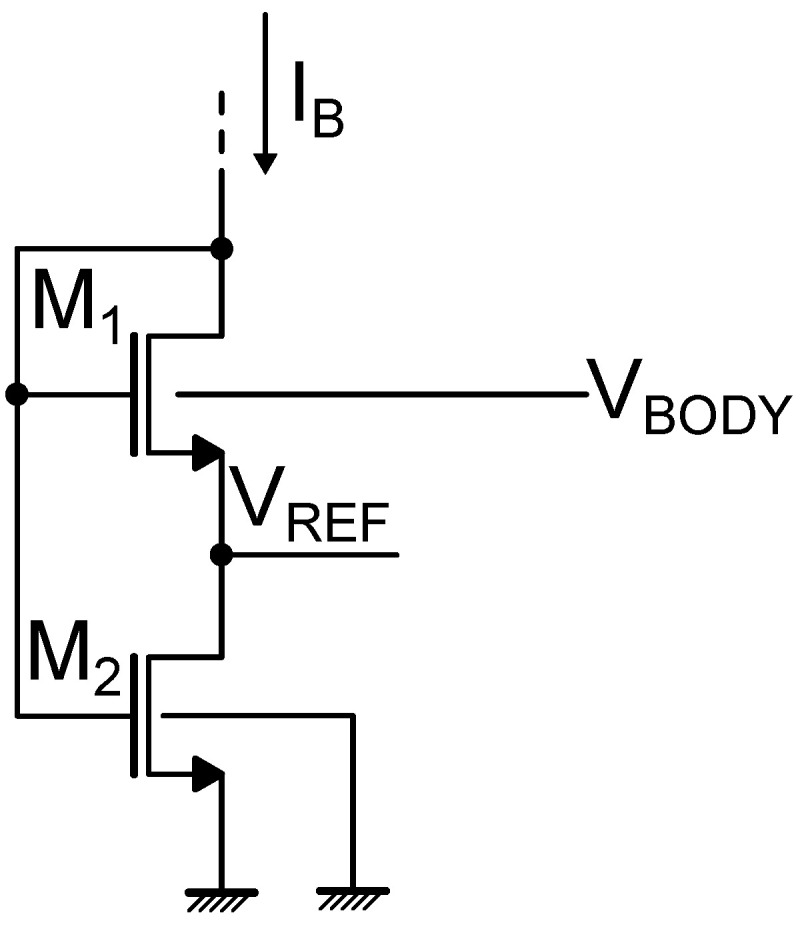
Schematic representation of a threshold voltage-based reference circuit employing body biasing.

In the threshold voltage-based voltage references implementations discussed until now, first order temperature invariance of the reference voltage is obtained by proper selection of the transistor types and appropriate sizing of the devices. However, there exists also the possibility to realize temperature compensation by acting on the transistor body biasing [28,29,30,31], as illustrated by the circuit shown in Figure 7. When a transistor features a source-to-body voltage ($V_{SB}$) different from zero, its threshold voltage is given by

(33) $$V_{TH}=V_{TH0}+\gamma \left(\sqrt{2\Phi_{F}+V_{SB}}-\sqrt{2\Phi_{F}}\right)\approx V_{TH0}+\left(n-1\right)V_{SB}$$

where $V_{TH0}$ is the threshold voltage with zero body biasing, γ the body effect constant, and $\Phi_{F}$ the Fermi potential [28]. Considering the circuit illustrated in Figure 7, the reference voltage is expressed as

(34) $$V_{REF}=V_{GS2}-V_{GS1}=\Delta V_{GS}^{\prime }+f\left(V_{BODY}\right)=nV_{T}ln\left(\frac{K_{1}}{K_{2}}\right)+f\left(V_{BODY}\right)$$

where $\Delta V_{GS}^{\prime }$ corresponds to the gate-to-source voltage difference between $M_{2}$ and $M_{1}$ when the substrate of $M_{1}$ is connected to ground (i.e., $nV_{T}ln\left(\frac{K_{1}}{K_{2}}\right)$ with $K_{i}$ the aspect ratio of $M_{i}$, supposing that the two devices are of the same type and share the same threshold voltage), and *f* is a function of the body biasing, whose detailed expression can be found in [28]. By adjusting $V_{BODY}$ and the transistors size, a temperature independent reference voltage, at the first order, can be obtained.

As for bandgap-like voltage reference circuits, higher order effects affecting the temperature dependence are present also in threshold voltage-based solutions and must be taken into account in order to improve (i.e., lower) the TC.

## 3. MOSFET-Based Voltage References: State-of-the-Art

A collection of MOSFET-based voltage references circuits from the last decade, making up the current state-of-the-art scenario, is presented in Table 1, reporting for each implementation found in the literature the most significant parameters, if available, including year of publication, employed technology (Tech.), reference (Ref.), type (BG = bandgap-like, VTH = threshold voltage-based, or other), minimum supply voltage (Min. $V_{DD}$), mean TC, minimum power consumption (Min. $P_{Cons}$), temperature (Temp.) range, $\frac{\sigma }{\mu }$ to quantify the spread, area, LS, PSR at 100 Hz, and the utilization of trimming.

As evident from Table 1, in recent years the employment of fully bandgap-like based implementations has been rather limited. The threshold voltage-based solutions are preferred as they allow lower supply voltage as well as power consumption. Hybrid architectures, relying on both bandgap-like and threshold voltage-based techniques, or other solutions, employing different approaches, have also emerged.

Concerning fully bandgap-like (BG) solutions, two main lines of research can be identified: one focuses on providing novel ways of implementing higher-order temperature compensation in addition to traditional architectures, while the other aims at proposing innovative overall circuit structures. When considering the first line of research, an example is provided by [32,56], which proposes to achieve second-order temperature compensation in a traditional Brokaw circuit by using the different temperature properties of the resistor types available in the technology, in particular P+ diffusion and poly resistors [32], and resistive P-poly and N-poly resistors [56], respectively. The second line of research [6] proposes a novel current-mode circuit topology that facilitates the amplifier design, since the input common mode is very low and the required output is about half of the supply voltage, thus relaxing the supply voltage limit and enabling operation with a supply as low as 0.4 V. Moreover, to cancel the term determining the non-linearity of the generated CTAT current, its equal and opposite replica current is injected in the proper node. In addition to the solution discussed in [6], another interesting innovative approach is proposed in [59], where a time-domain-based architecture for implementing the voltage reference is employed. The PTAT voltage is generated by subtracting the gate-to-source voltages produced during two consecutive phases by the same diode-connected NMOS transistor, biased with different currents in each phase and operating in weak inversion. As the same transistor is used in both phases, mismatch effects are inherently reduced for the PTAT voltage generation. The obtained PTAT voltage is then combined with a CTAT voltage by means of a switched capacitor difference amplifier. This solution, including a 5-bit trimming circuit, allows achieving a sub-ppm/°C TC, as proven through Montecarlo simulation results.

Concerning threshold voltage-based (VTH) reference solutions, several implementations can be found including ZTC-based architectures [17,38], 2T and 3T structures [26,27,47], self-cascode MOSFET [21,22,28,39,49,51,63], body biasing [4,28,29,30,31,54,55], and self-biasing [2,21,43,45,49,51,52,61]. As explained in Section 2, the basic VTH circuit consists of a current generator and active load, with the latter that is typically a diode-connected transistor [4,20,27,31,34,47] a stack of diode-connected transistors [37,54], a SCM [22,23,28,39] or a variation in the reported implementations, for example including modifications to realize self-biasing [21,38,45,49,51] start-up [33,63] or temperature compensation features [17,43,52,61]. The required current generator can be realized in a wide range of ways: leakage-based solutions employing transistors in cut-off [4,23,26,27,31,47,54,55], bulk-driven architectures [28], proportional-to-μT^2^ current generators [33,34] achieved by translating a PTAT voltage into a current by means of transistors in strong inversion, modified standard PTAT current generators with gate leakage transistor as equivalent resistor [50], improved triode-based Widlar references [43]. For what concerns the required active load implementation, instead, a single diode-connected transistor is employed in 2T and 3T voltage references, where the ZTC condition is achieved by employing transistors of different types (e.g., PMOS and NMOS [27,47]) or with different size [26]. If a rather high reference voltage is required, the 2T/3T configuration can be expanded in order to include multiple stacked diode-connected transistors as load [37]. SCM structures and their modifications to include self-biasing features are also quite common realizations for active loads in VTH-based voltage references and different implementations can be identified. Typically different types of transistors (thin-oxide and thick-oxide) are employed [21,39,49,51,63]; however, also the same type of transistors can be used when obtaining different threshold voltages by means of body biasing [28,29] or different transistor sizes [33], e.g., relying on the reverse short channel and narrow width effects [22].

SCM structures are also common in hybrid solutions, which combine a VTH-based approach with the use of an operational amplifier [13,40,41,42,46]; in these cases, the SCM architecture, with proper transistor sizing and selection, is typically used for realizing a PTAT contribution [40,41,42]. Moreover, two-stage [52,61,64] and multi-stage [43] SCM circuits are also used, both in fully SCM-based and in hybrid solutions, for increasing the reference voltage and reducing process dependencies.

In addition to hybrid solutions employing SCM circuits, other voltage reference implementations that do not fit in the bandgap-like or VTH-based realizations also exist. Among these, Ref. [15] is particularly interesting as it exploits the action of an operational amplifier to produce a reference voltage that is an attenuated version of the ZTC bias voltage point; moreover, the proposed architecture implements the TC optimization by adjusting the transistor $V_{DS}$ by means of resistor trimming.

Another peculiar implementation is described in [35], where low TC is achieved by compensating the CTAT voltage, generated by a diode-connected NMOS, with a two-stage PTAT voltage generator, realized by an asymmetrical differential cell with two additional cross-coupled NMOS/PMOS pairs, which significantly enhance the slope of the PTAT voltage.

A common implementation among voltage reference circuits that do not fit in the bandgap-like or VTH-based realizations relies on the use of switched capacitor structures. They can be used for implementing weighted averaging between two voltages [36,60], for boosting the open loop gain of an equivalent common source amplifier to reduce the residual error on the generated reference voltage [65], or for supporting the sampling operation in a capacitively biased voltage reference core [66]. This last type of voltage reference implementation is very recent and proposes a novel ZTC biasing approach, relying on the current generated by a discharging capacitor for biasing a SCM structure featuring a thick-oxide and a thin-oxide transistor. The operation occurs according to two phases: during the reset phase the capacitance is charged to the supply voltage, whereas during the subsequent discharge phase the capacitance is discharged via the SCM devices, resulting in a time-varying output voltage. As the discharging current, after the initial transient is solely determined by the exponential I-V characteristic of the devices operating in weak inversion, decreases, the temperature dependency of the output voltage changes from PTAT to CTAT, and a near zero-TC point exists at a certain optimum time in the middle. By sampling the output voltage at the optimum time and employing an auto-zero buffer with high input impedance to drive an external load, a low-TC voltage reference is implemented.

The use of a buffer for enabling sufficient current drive capability is fundamental also in the automotive field, as detailed in [67], which proposes a voltage reference solution fabricated in 160 nm BCD technology, tailored to address the specifications for automotive applications, including operation across the industrial temperature range, a wider supply voltage range accommodating up to 5 V, and a high current drive capability reaching several microamperes.

Although the low-TC is often the main parameter of interest when evaluating voltage reference circuits, particular attention should be paid to LS and PSR as well, since independence from the supply voltage variations is also a feature of interest. In order to improve this feature, different solutions can be found among the reported works: traditional cascode structures [21,52] providing shielding from supply voltage variations; self-regulating MOS devices [27,47] acting as cascode structures to increase the effective output impedance of the current source in leakage-based solutions; native (i.e., zero-threshold-voltage) NMOS devices [22,38,57] which eliminate additional parasitic coupling paths due to n-well, thus, improving disturbances coupled through the power supply; self-regulating circuits [45]; pre-regulators [53] to suppress supply voltage ripples and provide a stable supply for the core reference circuits, MOSFET low-pass filters [33,34] for ensuring wide bandwidth PSR; and DIBL (drain-induced-barrier-lowering) compensation circuits [2,49,63].

In addition to the discussed parameters of interest, robustness against process variations must also be ensured. This can be achieved by means of standard trimming techniques [2,3,15,20,21,28,29,31,33,34,35,36,37,44,49,50,53,56,58,59,65] or by design, as it is achieved in [57], where two VTH-based voltages with opposite process and temperature characteristics are combined, or in [55], where optimum body selection is introduced to reduce the impact of threshold-voltage process variations.

Considering the reported works in Table 1, a general research trend is driving towards supply voltage and power consumption minimization, thus addressing the requirements of the applications enabling our increasingly “smarter” lifestyle; in order to fulfill the low-power consumption system specifications, the possibility to combine multiple voltage references [3,51] or current and voltage references [46,64] together in a single circuit has also emerged. The targeted power consumption indeed has moved from the microwatt domain of BJT-based implementations, to the nanowatt in early MOSFET-based solutions, and finally to the sub-nanowatt [2,21,26,31,47,50,54,55,57,63] and even sub-picowatt [22,23,27] power consumption in the last decade.

**Figure 8:**
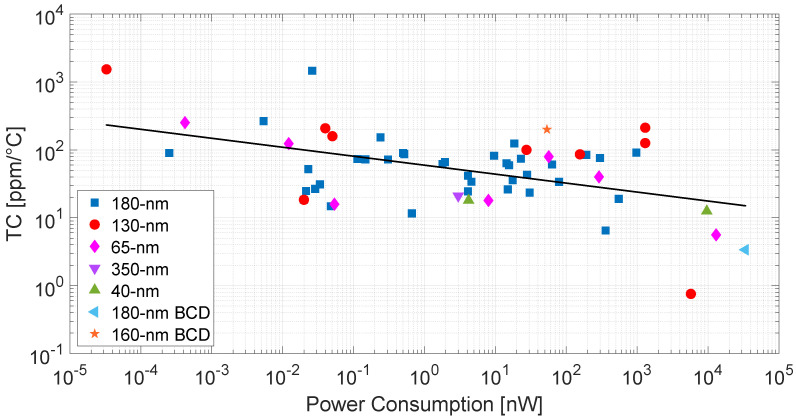
Graphical summary of the TC of the reported works as a function of power consumption. The representation is in logarithmic scale.

As shown in Figure 8, which reports the TC as a function of the power consumption, a correlation between the two can be noticed; the lower power consumption usually implies larger TC, thus imposing a trade-off. This trend is justified by the fact that, to reduce power consumption, MOSFET-based voltage references typically rely on subthreshold or leakage-based operations, which are more sensitive to temperature; hence, the lower is the power consumption, the larger are the effects due to temperature variations [44]. A correlation is not found instead between power consumption and LS/PSR. This can be expected as the robustness against supply voltage variations is achieved by appropriate design techniques which do not necessarily imply additional power consumption (e.g., the addition of native MOS devices or cascode structures on an existing circuit branch).

One of the main challenges that future research has to address, is proposing MOSFET-based voltage references able to overcome the trade-off between power consumption and TC. Moreover, as shown in Table 1, almost all MOSFET-based solutions are implemented in old technological nodes (350 nm, 180 nm, and 130 nm), another area open for innovation in the field is given by implementing voltage reference circuits in more advanced processes (65 nm, 40 nm, and lower), thus addressing the progressive technological scaling.

## 4. Conclusions

This paper has presented an overview on MOSFET-based reference voltage circuit implementations, illustrating their general principles of operation. Furthermore, the circuit solutions proposed in the last decade have been summarized, highlighting their main characteristics and peculiarities, in order to provide a detailed outline of the state-of-the-art, which can act as a starting point for future research endeavors in the field. In particular, the main current trends that can be identified involve minimizing the power consumption, supporting lower supply voltages to address the requirements of portable, wearable and implantable applications, and abandoning bandgap-like solutions in favour of VTH-based or other (e.g., hybrid) voltage reference implementations. Concerning future research endeavors, the development of MOSFET-based solutions in scaled nodes (e.g., 65 nm, 40 nm, and lower) surely constitutes a subject of interest, as the majority of proposed implementations is realized in older and well-established processes. This would allow MOSFET-based references in the literature to finally match the technological advancements and therefore, address any challenge that could arise with scaled processes. Moreover, proposing and investigating solutions that allow overcoming the limits of available MOSFET-based reference solutions would also constitute a subject of great interest. In particular in order to make MOSFET-based references more appealing, the trade-off between power consumption and TC should be broken, possibly with the aid of appropriate trimming strategies, that minimize power consumption, area occupation, and cost (e.g., in terms of employed temperature points).

## Figures and Tables

**Table 1:** Summary of state-of-the-art MOSFET-based voltage references.

Work	Year	Tech.	Ref. Type	Min. $V_{\mathrm{DD}}$ [V]	$V_{\mathrm{REF}}$ [mV]	Mean TC [ppm/°C]	Min. $P_{\mathrm{Cons}}$ [nW]	Temp. Range [°C]	$\frac{\sigma }{\mu }$ [%]	Area [mm^2^]	LS [%/V]	PSR (100 Hz) [dB]	Trim.
[26]	2015	180	VTH	0.15	17.69	1462.4	0.0261	0 to 120	1.6	0.0012	2.03	−64	NA
[32]	2015	65	BG	0.75	474	40	290	−40 to 90	3.37	0.0198	0.2423	−40	NA
[28]	2015	180	VTH	0.4	118.46	63.6	14.4	−40 to 125	0.6	0.012	NA	−44.2	Yes
[6]	2017	180	BG	0.4	212.4	84.5	192	−40 to 130	NA	0.09	0.957	−40	NA
[29]	2016	180	VTH	0.45	118.41	59.4	15.6	−40 to 85	0.58	0.0132	0.033	−50.3	Yes
[33]	2017	180	VTH	1.1	893	19	550	−30 to 80	0.185	0.018	0.093	−75	Yes
[34]	2017	180	VTH	0.8	489	6.5	360	−30 to 110	0.5	0.018	0.076	−75	Yes
[15]	2017	65	Other	0.8	428	5.6	13000	−40 to 125	5.3	0.0104	0.1	−87	Yes
[20]	2017	350	VTH	0.9	710	21	3	−20 to 80	12.29	0.068	0.26	NA	Yes
[35]	2018	180	Other	1	756	74	23	−40 to 125	0.95	0.01615	0.524	−52	Yes
[36]	2017	180	Other	1.8	350.8	76	300	0 to 75	0.11	0.023	0.03	NA	Yes
[37]	2017	180	VTH	1.4	1.25	31	0.0336	0 to 100	NA	0.025	0.31	−41	Yes
[38] *	2017	130	VTH	1.2	450	86	156	−55 to 125	6.5	0.006	NA	−89	NA
[39]	2017	180	VTH	0.8	328	33.8	79	10 to 100	NA	0.014	0.21	−55	NA
[27]	2017	65	VTH	0.4	342.8	252.2	0.00042	−40 to 60	4.9	0.000104	0.47	NA	NA
[40]	2018	180	Other	1.1	755	34	4.6	−15 to 140	0.64	0.0598	0.28	−9	NA
[21] SBSCM	2018	180	VTH	0.45	256.6	72.4	0.147	0 to 120	NA	0.002	0.15	−43.9	Yes
[21] SBNMOS	2018	180	VTH	0.6	457.1	11.6	0.664	0 to 120	NA	0.0017	0.11	−46.8	Yes
[41]	2018	180	Other	0.4	210	82	9.6	−40 to 140	0.31	0.021	0.027	−48	NA
[42]	2018	180	Other	0.6	218.3	23.5	30.5	−40 to 125	0.45	0.075	0.4	−47.5	NA
[43]	2018	130	VTH	1.1	800	100	27.5	−40 to 85	NA	0.003	2	NA	No
[22] 3T	2018	130	VTH	0.3	26	208	0.04	−25 to 125	NA	0.0006	0.188	−67.3	No
[22] 4T	2018	130	VTH	0.4	27.2	159	0.0504	−25 to 125	NA	0.0012	0.08	−98	No
[22] Sub-$V_{T}$	2018	130	VTH	0.12	8.42	1537	0.0000328	0 to 125	NA	0.0009	1.62	−38.4	No
[44]	2019	40	Other	0.9	583	18	4.2	−40 to 120	0.204	0.01	0.23	−72	Yes
[45]	2019	180	VTH	0.4	151	89.83 ^[*a*]^	1	−40 to 125	0.84 ^[*a*]^	0.005	0.163 (0.0154 ^[*a*]^)	NA	NA
[17] A	2019	130	VTH	0.45	355	126	1300	−40 to 80	9.58	0.017	4.7	−43	NA
[17] B	2019	130	VTH	0.45	315	212	1300	−40 to 80	3.17	0.017	1.9	−46	NA
[46]	2019	180	Other	0.7	368	43.1	28	−40 to 125	0.35	0.055	0.027	−43	NA
[47]	2019	65	VTH	0.5	210.1	123.7	0.0122	0 to 100	4.6	0.003256	0.3	NA	No
[48]	2020	180	Other	1.2	500	NA	6120	0 to 100	NA	0.073	0.28	−42	NA
[49]	2020	180	VTH	0.34	147.9	14.8	0.048	0 to 100	NA	0.0332	0.019	−65	Yes
[50]	2020	130	VTH	1	NA	18.4	0.02	−25 to 85	NA	0.003	0.15	−50	Yes
[13]	2020	180	Other	0.5	151.9	35.7	17.6	−40 to 85	NA	0.0092	0.09	−51.8	No
[51] VREF1	2020	180	VTH	0.6	332	41.67	4.12	−40 to 125	0.527	0.0108	0.0505	NA	NA
[51] VREF2	2020	180	VTH	0.8	660	24.48	4.12	−40 to 125	0.423	0.0108	0.114	NA	NA
[52]	2021	180	VTH	0.9	261	62	1.8	−40 to 130	0.43	0.0059	0.013	−73.5	No
[23] *	2021	180	VTH	0.12	65.7	89.81	0.000252	−40 to 120	10.9	0.00007	0.22	−61	No
[53]	2021	180	Other	1.5	985	60.86	63	NA	NA	0.015	0.003	−80	Yes
[54]	2021	180	VTH	0.25	91.4	265	0.0054	0 to 120	0.56	0.0022	0.16	−70	NA
[30]	2021	180	VTH	0.25	118.1	73.5	0.113	−40 to 140	1.1	0.0009	0.3	−65	NA
[31]	2022	180	VTH	0.6	350	72.17	0.306	−40 to 80	0.58	0.022	0.093	−39	Yes
[55] VR1	2022	180	VTH	0.6	431	52	0.023	0 to 85	0.35	0.003	0.03	−47	No
[55] VR2	2022	180	VTH	0.8	620	153	0.24	0 to 85	0.9	0.003	0.033	−44	No
[56] *	2022	40	BG	1.2	800	12.51	9600	−40 to 90	0.75		0.028	−71.69	Yes
[57]	2023	180	VTH	0.5	288	90	0.5	−10 to 100	0.574	0.0029	0.23	NA	Yes
[58]	2023	65	VTH	0.4	107.2	79.4	56.7	−20 to 80	NA	0.0084	0.54	−66.5	Yes
[59] *	2023	130	BG	1.8	993.6	0.756	3210 5730 ^[*b*]^	−40 to 100	1.43	0.0767 0.1482 ^[*b*]^	NA	NA	Yes
[2] *	2023	180	VTH	0.6	307.8	24.8	0.0214	−20 to 80	NA	0.003	0.02	−54	Yes
[60] *	2023	180	Other	0.9	266	90.95	976	−30 to 120	NA	0.701	NA	NA	NA
[61]	2024	180	VTH	0.8	293.5	66.1	1.95	−40 to 85	NA	0.004	0.011	−53	NA
[62] *	2024	180 BCD	Other	0.85	508.3	3.38	33700	−65 to 225	NA	0.00676	NA	NA	NA
[63]	2024	180	VTH	0.6	317.6	86.6	0.52	0 to 100	NA	0.0016	0.016	−61.1	NA
[64]	2024	180	Other	0.8	270	124	18.51	−40 to 130	0.81	0.025	0.011	−66	NA
[65] *	2024	180	Other	0.5	237.2	26.2	15	−25 to 85	NA	0.072	NA	NA	Yes
[66]	2024	65	Other	0.7	204.1	18	8	−40 to 85	0.78	0.00315 (only core)	NA	−75	NA
[67]	2024	160 BCD	Other	1.2	390	200	53.52	−40 to 125	NA	0.087 (only core)	0.137	−83	NA
[4] *	2024	180	VTH	0.5	195.5	26.7	0.0288	0 to 100	NA	0.00236	0.0017	−50	NA
[3] *	2024	65	VTH	0.45	276	15.86	0.05383	−10 to 155	NA	0.00348	0.077	−56.3	Yes

* Simulation results. ^[*a*]^ at 1.8-V supply voltage. ^[*b*]^ including buffer.
